# Pretreatment with NaCl Promotes the Seed Germination of White Clover by Affecting Endogenous Phytohormones, Metabolic Regulation, and Dehydrin-Encoded Genes Expression under Water Stress

**DOI:** 10.3390/ijms19113570

**Published:** 2018-11-12

**Authors:** Yiqin Cao, Linlin Liang, Bizhen Cheng, Yue Dong, Jiaqi Wei, Xiaolan Tian, Yan Peng, Zhou Li

**Affiliations:** Department of Grassland Science, College of Animal Science and Technology, Sichuan Agricultural University, Chengdu 611130, China; caoyiqin429@163.com (Y.C.); LiangllCLJ@163.com (L.L.); Chengbizhengrass@163.com (B.C.); D13981799690@163.com (Y.D.); 13837290645@163.com (J.W.); 15680830256@163.com (X.T.); pengyanlee@163.com (Y.P.)

**Keywords:** oxidative damage, antioxidant defense, osmotic adjustment, growth, gene expression

## Abstract

This study was designed to examine the effects of NaCl pretreatment on the seed germination of white clover (*Trifolium repens* cv. Ladino) under water stress induced by 19% polyethylene glycol (PEG) 6000. Lower concentrations of NaCl (0.5, 1, and 2.5 mM) pretreatment significantly alleviated stress-induced decreases in germination percentage, germination vigor, germination index, and radicle length of seedlings after seven days of germination under water stress. The soaking with 1 mM of NaCl exhibited most the pronounced effects on improving seed germination and alleviating stress damage. NaCl-induced seeds germination and growth could be associated with the increases in endogenous gibberellic acid (GA) and indole-3-acetic acid (IAA) levels through activating amylases leading to improved amylolysis under water stress. Seedlings pretreated with NaCl had a significantly lower osmotic potential than untreated seedlings during seed germination, which could be related to significantly higher soluble sugars and free proline content in NaCl-treated seedlings under water stress. For antioxidant metabolism, NaCl pretreatment mainly improved superoxide dismutase, peroxidase, ascorbate peroxidase, and glutathione reductase activities, transcript levels of *FeSOD*, *APX*, and *DHAR*, and the content of ascorbic acid, reduced glutathione, and oxidized glutathione during seed germination under water stress. The results indicated that seeds soaking with NaCl could remarkably enhance antioxidant metabolism, thereby decreasing the accumulation of reactive oxygen species and membrane lipid peroxidation during germination under water stress. In addition, NaCl-upregulated dehydrin-encoded genes *SK2* expression could be another important mechanism of drought tolerance during seeds germination of white clover in response to water stress.

## 1. Introduction

With the intensification of the global greenhouse effect, abnormal climates such as persistent high temperatures or drought are increasing worldwide. Drought has become one of the major abiotic stresses affecting plant growth and development [1]. For improving drought tolerance of plant species, two hot and main methods are widely studied: one is transgenic technology via the transformation of stress-defensive genes into plants; another is the utilization of plant growth regulators (PGRs) during plant growth and development [2,3,4]. Previous studies have shown that transferring a single stress-resistant gene into plants can improve stress tolerance in the laboratory, but the effects of transgenosis in the field are difficult to estimate, and the cultivation of transgenic plants is often also limited, due to transgenosis-caused possible environmental risks in many countries. Comparatively, the application of PGRs on enhancing stress tolerance in plants has multiple advantages, including low costs, simple operation, and stable effects [5]. PGRs such as trinexapac-ethyl, glycine, and chitosan have been widely used for the improvement of drought tolerance in crops and grass species [6,7,8].

It is well known that the excessive accumulation of Na^+^ in soil is the main cause of salt stress [9]. Salt stress limits water uptake and leads to an excessive accumulation of Na^+^ in plants. The overaccumulation of Na^+^ disrupts the metabolic homeostasis, and also inhibits the absorption of other ions, resulting in plant growth inhibition and death [10]. However, as an important inorganic osmolyte in plants, Na^+^ plays a critical role in osmotic adjustment (OA) when plants suffer from abiotic stress [11]. The study of Wang et al. (2004) [12] found that succulent xerophytes *Haloxylon ammodendron* and *Zygophyllum xanthoxylum* accumulated Na^+^ as effective inorganic osmolytes for the adaptation to drought stress. Lower concentration of exogenous Na^+^ also can effectively improve drought tolerance in plants. For example, exogenous applications of NaCl (50 mM) enhanced osmotic adjustment, antioxidant defense, and photosynthesis in the halophyte species *Atriplex halimus*, associated with improved stress tolerance under water stress condition [13]. Our recent study also showed that pretreatment with 50 mM of NaCl could significantly promote OA, water use efficiency, and photosynthesis, contributing to enhanced drought tolerance in white clover, and NaCl-pretreated white clover also maintained better growth than untreated plants under normal and drought conditions [14]. These findings thus indicate that the positive function of Na^+^ is more than OA in plants.

White clover (*Trifolium repens*) is one of the most important leguminous forages, with higher crude protein, nutrient elements, and strong nitrogen fixation abilities. Due to those fine characteristics, white clover is widely cultivated in many advanced animal husbandry countries, such as the United States, Australia, and New Zealand. White clover is also used as turf or ground cover grass throughout South China and other countries [15,16,17]. However, white clover only adapts to cold and humid climates, and it exhibits a lower drought tolerance than other leguminous grasses such as alfalfa (*Medicago sativa*) [18]. Water deficit more easily to decreases yield and quality of white clover, thereby limiting its cultivation and utilization in many areas. In response to drought stress, seed germination is the most sensitive stage. It has been reported that priming with PGRs such as mannose, mannitol, ascorbic acid, and spermidine could significantly improve the seed germination of various plant species under drought or water stress conditions [19,20,21]. However, it is not well documented whether NaCl could act as an important PGR in regulating seeds germination under drought stress or not. The NaCl priming-induced stress tolerance mechanism also needs to be further investigated during seed germination.

Objectives of this study were to investigate effects of priming with NaCl on seed germination characteristics and to further reveal whether NaCl-priming-regulated stress tolerance is associated with key metabolic processes, including changes of endogenous phytohormones, starch metabolism, OA, antioxidant defense, and dehydrin-encoded genes expression during the seed germination of white clover under normal and water stress conditions.

## 2. Results

### 2.1. Effects of Seed Soaking with NaCl on Germination Characteristics 

**Figure 1:**
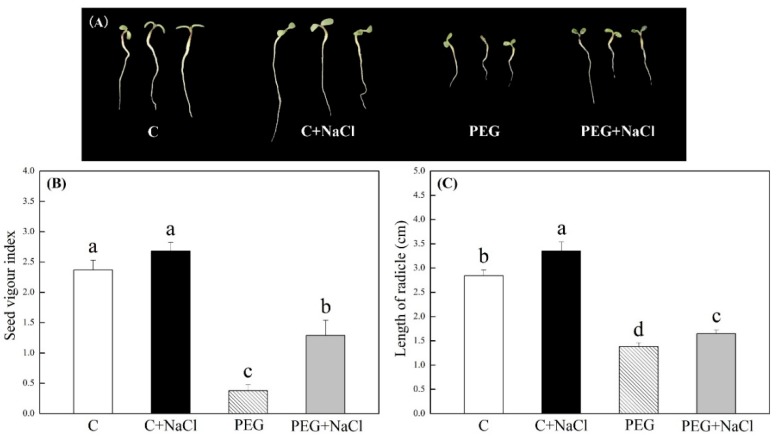
Effects of seeds soaking with water or NaCl (1 mM) on (**A**) phenotypic change, (**B**) seed vigor index (VI), and (**C**) length of radical after seven days of germination under normal condition and water stress induced by 19% polyethylene glycol (PEG) 6000. Vertical bars indicate the ±SE of mean (*n* = 6). Different letters above columns indicate significant difference (*p* ≤ 0.05).

Under normal conditions, different concentrations of NaCl (0, 0.25, 0.5, 1, 2.5, 5, and 10 mM) pretreatment did not significantly affect the germination percentage (GP), germination vigor (GV), germination index (GI), and mean germination speed (MGS) of white clover seeds during seven days of germination (Table 1). A concentration of 19% polyethylene glycol (PEG)-induced water stress significantly increased MGS and decreased GP, GV, and GI. Seeds priming with 0.5, 1, and 2.5 mM of NaCl could evidently improve GP, GV, and GI, and decrease MGS during germination under water stress. Seeds soaking with 1 mM of NaCl had the most pronounced effects on increases in GV, GP, and GI, and on the decrease of MGS under water stress. However, lower (0.25 mM) and higher (5 and 10 mM) concentrations of NaCl priming did not show positive effects on GP, GV, GI, and MGS under water stress (Table 1). After seven days of germination, the phenotypic change of seedlings was showed in Figure 1A when seeds were pretreated with and without 1 mM of NaCl. NaCl-pretreated seedlings (1 mM) had 240% significantly higher seed vigor index (VI) than untreated seedlings under water stress (Figure 1B). Seeds soaking with 1 mM of NaCl also exhibited 17.9% and 16.0% higher radicle lengths, as compared to seeds soaking with water after seven days of germination under normal and water stress conditions, respectively (Figure 1C).

### 2.2. Effect of Seed Soaking with NaCl on the Changes of Endogenous Phytohormones

**Figure 2:**
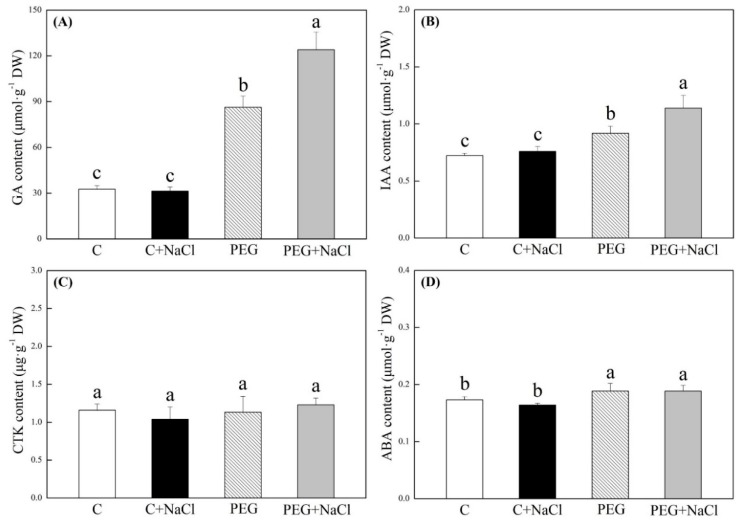
Effects of seeds soaking with water or NaCl (1 mM) on (**A**) gibberellin (GA) content, (**B**) indole-3-acetic acid (IAA) content, (**C**) cytokinin (CTK) content, and (**D**) abscisic acid (ABA) content after seven days of germination under normal conditions and water stress induced by 19% polyethylene glycol (PEG) 6000. Vertical bars indicate ±SE of the mean (*n* = 6). Different letters above columns indicate significant differences (*p* ≤ 0.05).

The content of gibberellin (GA), indole-3-acetic acid (IAA), cytokinin (CTK), and abscisic acid (ABA) in seedlings are not significantly affected by NaCl pretreatment under normal conditions (Figure 2). The water stress significantly increased the content of GA and IAA content in seedlings with or without NaCl pretreatment relative to the normal condition; and seeds soaking with 1 mM of NaCl exhibited 43.7% and 24.1% higher GA and IAA contents, as compared to seeds soaking with water after seven days of germination under water stress, respectively (Figure 2A,B). CTK content showed no difference between NaCl-pretreated and untreated seedlings under normal and water stress conditions (Figure 2C). The water stress increased endogenous ABA content in seedlings after seven days of germination, but ABA content did not show any significant difference between NaCl-pretreated and untreated seedlings under water stress conditions (Figure 2D).

### 2.3. Effect of Seed Soaking with NaCl on Starch Metabolism and Osmotic Adjustment

**Figure 3:**
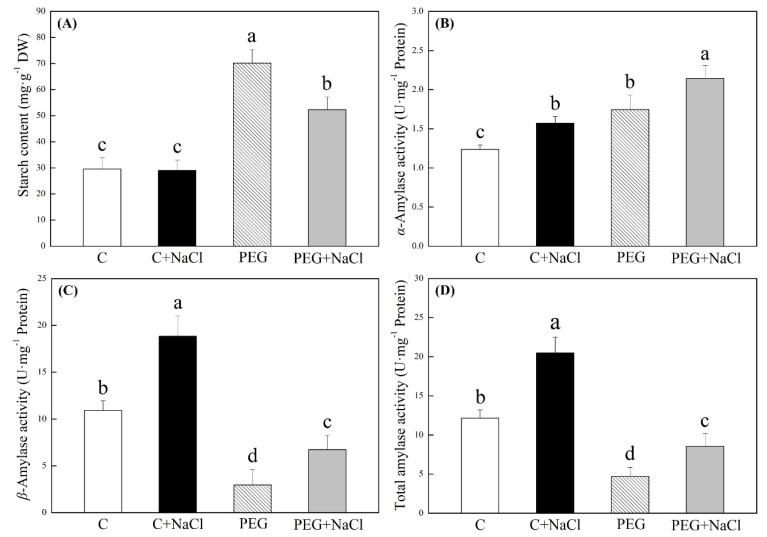
Effects of seeds soaking with water or NaCl (1 mM) on (**A**) starch content, (**B**) α-amylase activity, (**C**) β-amylase activity, and (**D**) total amylase activity after seven days of germination under normal conditions and under water stress induced by 19% polyethylene glycol (PEG) 6000. Vertical bars indicate ±SE of mean (*n* = 6). Different letters above columns indicate significant differences (*p* ≤ 0.05).

**Figure 4:**
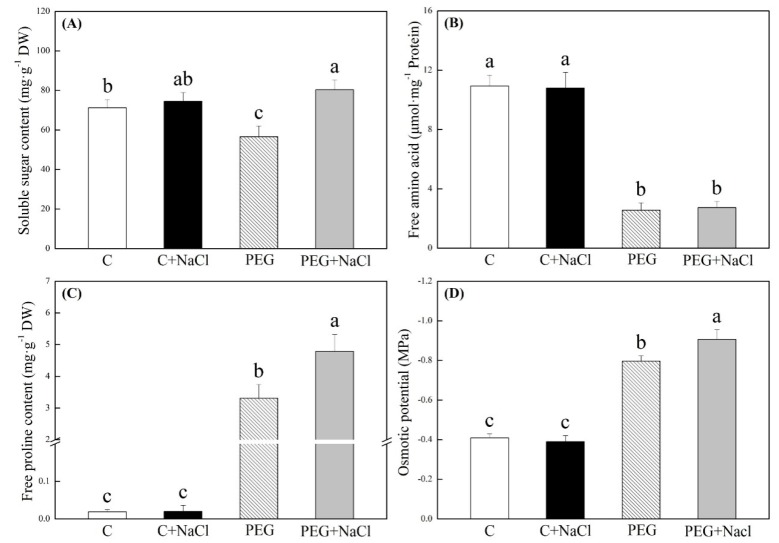
Effects of seeds soaking with water or NaCl (1 mM) on (**A**) soluble sugar, (**B**) free amino acid, (**C**) free proline, and (**D**) osmotic potential after seven days of germination under normal condition and water stress induced by 19% polyethylene glycol (PEG) 6000. Vertical bars indicate ±SE of mean (*n* = 6). Different letters above columns indicate significant difference (*p* ≤ 0.05).

In spite of NaCl priming, water stress significantly inhibited starch degradation, leading to higher starch content in water-stressed seedlings relative to non-stressed seedlings (Figure 3A). Starch content showed no significant difference between the NaCl-pretreated and untreated seedlings under normal conditions, but exogenous NaCl priming significantly accelerated amylolysis in seedlings under water stress (Figure 3A). The α-amylase activity was significantly increased by an exogenous application of NaCl under normal and water stress conditions (Figure 3B). In addition, seeds soaking with NaCl significantly improved β-amylase and total amylase activities under normal conditions and water stress (Figure 3C,D). The soluble sugar, free amino acids, free proline, and osmotic potential in seedlings are not significantly affected by NaCl pretreatment under normal conditions (Figure 4). The NaCl-pretreated seedlings significantly exhibited 42% and 45% higher soluble sugar and free proline contents than untreated seedlings under water stress (Figure 4A,C). Exogenous NaCl pretreatment also further decreased osmotic potential (OP) in seedlings under water stress, and seeds soaking with NaCl had 11.5% significantly lower OP than seeds without NaCl pretreatment after seven days of germination in response to water stress (Figure 4D).

### 2.4. Effect of Seed Soaking with NaCl on Oxidative Damage

**Figure 5:**
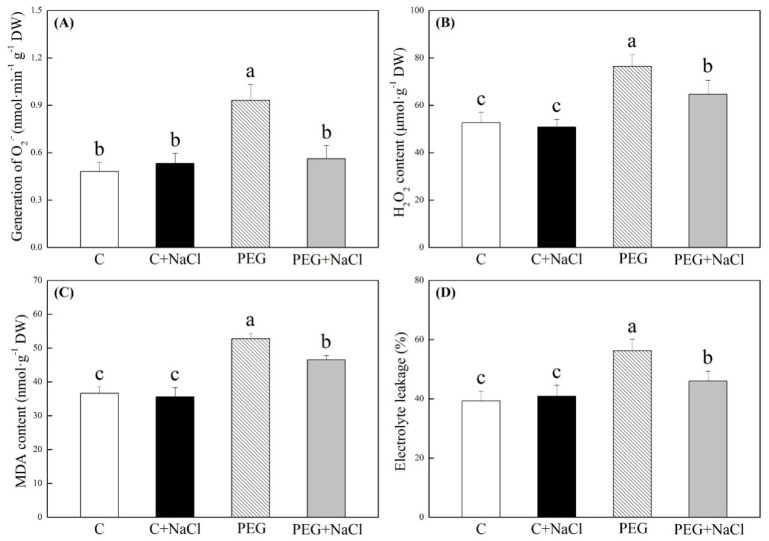
Effects of seeds soaking with water or NaCl (1 mM) on (**A**) the generation of superoxide anions (O_2_^−^), (**B**) hydrogen peroxide (H_2_O_2_), (**C**) malondialdehyde (MDA) content, and (**D**) electrolyte leakage after seven days of germination under normal conditions and under water stress induced by 19% polyethylene glycol (PEG) 6000. Vertical bars indicate the ±SE of the mean (*n* = 6). Different letters above the columns indicate significant differences (*p* ≤ 0.05).

Water stress significantly induced the accumulation of the generation of superoxide anion (O_2_^−^), hydrogen peroxide (H_2_O_2_), and malondialdehyde (MDA) in seedlings under water stress (Figure 5A–C). O_2_^−^, H_2_O_2_, MDA, and electrolyte leakage (EL) showed no differences between NaCl-pretreated and untreated seedlings under normal water conditions (Figure 4). After seed soaking with NaCl, the O_2_^−^ content in seedlings decreased by 32% in response to water stress (Figure 5A). Similarly, the NaCl-pretreated seedlings had 15, 12, and 18% significantly lower H_2_O_2_, MDA, and EL than untreated seedlings under water stress (Figure 5B–D). All results showed that seeds soaking with NaCl effectively alleviated oxidative damage during seed germination under water stress. 

### 2.5. Effect of Seed Soaking with NaCl on Antioxidant Metabolism 

**Figure 6:**
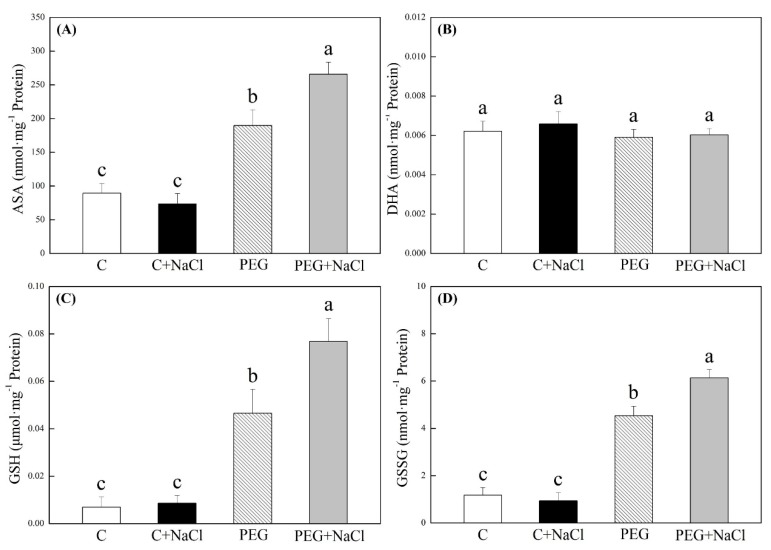
Effects of seeds soaking with water or NaCl (1 mM) on (**A**) ascorbic acid (ASA), (**B**) dehydroascorbic acid (DHA), (**C**) reduced glutathione (GSH), and (**D**) oxidized glutathione (GSSG) content after seven days of germination under normal conditions and water stress induced by 19% polyethylene glycol (PEG) 6000. Vertical bars indicate ±SE of mean (*n* = 6). Different letters above columns indicate significant differences (*p* ≤ 0.05).

**Figure 7:**
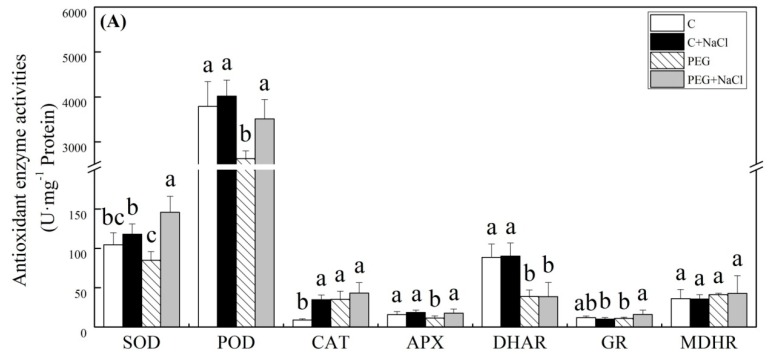

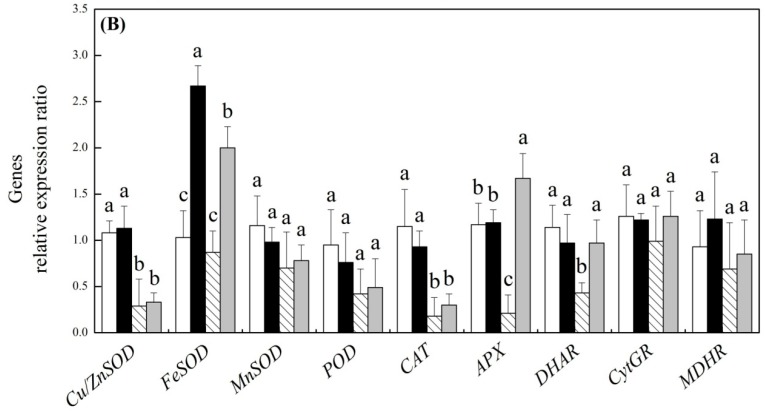
Effects of seeds soaking with water or NaCl (1 mM) on (**A**) antioxidant enzyme activities and (**B**) the relative expression ratio of genes encoding antioxidant enzymes after seven days of germination under normal conditions, and water stress induced by 19% polyethylene glycol (PEG) 6000. Vertical bars indicate ±SE of mean (*n* = 6). Different letters above columns indicate significant difference (*p* ≤ 0.05).

The contents of ascorbic acid (ASA), dehydroascorbic acid (DHA), reduced glutathione (GSH), and oxidized glutathione (GSSG) in seedlings were not significantly affected by the exogenous application of NaCl after seven days of germination under normal conditions (Figure 6). Water stress significantly enhanced the accumulation of ASA, GSH, and GSSG, but it did not induce DHA accumulation in seedlings. Exogenous NaCl pretreatment further improved stress-induced increases in ASA, GSH, and GSSG content in seedlings under water stress (Figure 6A,C,D). For changes of antioxidant enzyme activities, seeds soaking with NaCl had stimulative effects on superoxide dismutase (SOD), guaiacol peroxidase (POD), ascorbate peroxidase (APX), and glutathione reductase (GR) activities under water stress. A 72, 34, 56, or 47% higher SOD, POD, APX, or GR activity was observed in NaCl-pretreated seedlings as compared to that in untreated seedlings under water stress, respectively (Figure 7A). NaCl pretreatment also significantly improved CAT activity in seedlings under normal water conditions (Figure 7A). Under normal water conditions, transcript levels of all genes encoding antioxidant enzymes were not significantly affected by exogenous NaCl except *FeSOD* (Figure 7B). The NaCl-pretreated seedlings showed 2.6- and 2.3-fold higher transcript levels of *FeSOD* than untreated seedlings under normal and water stress conditions, respectively. Interestingly, water stress obviously inhibited the expression of *APX* and *DHAR* in seedlings without NaCl pretreatment, but exogenous NaCl significantly up-regulated *APX* and *DHAR* genes expression in seedlings under water stress (Figure 6B).

### 2.6. Effect of Seed Soaking with NaCl on Dehydrin-Encoded Gene Expression

**Figure 8:**
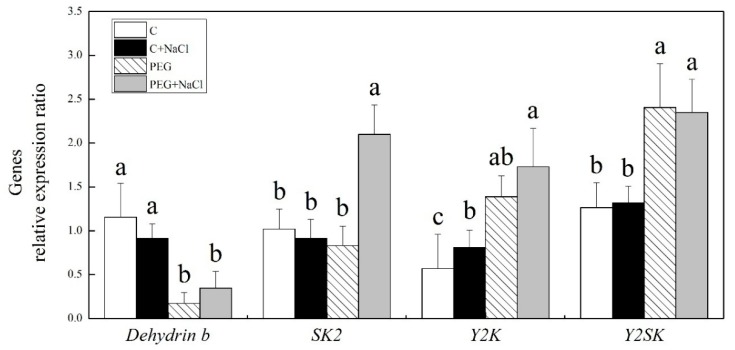
Effects of seeds soaking with water or NaCl (1 mM) on the relative expression ratio of genes encoding dehydrins after seven days of germination under normal conditions and water stress induced by 19% polyethylene glycol (PEG) 6000. Vertical bars indicate ±SE of the mean (*n* = 6). Different letters above columns indicate significant differences (*p* ≤ 0.05).

The transcript levels of four different genes (*SK2*, *SK2*, *Y2SK* and *dehydrin b*) encoding dehydrins were examined in this study (Figure 8). Under normal water conditions, exogenous NaCl had no significant influences on the transcript levels of all dehydrin-encoded genes. Water stress significantly inhibited *dehydrin b* expression, whereas it up-regulated the expression of *Y2K* and *Y2SK*, in spite of exogenous NaCl pretreatment. The transcript levels of *SK2* showed no change in seedlings without NaCl pretreatment in response to water stress. Seeds soaking with NaCl significantly increased the transcript level of *SK2* after seven days of germination under water stress. The transcript level of *SK2* in seedlings with NaCl pretreatment was 2.5 times higher than the transcript levels of this gene in seedlings without NaCl treatment under water stress (Figure 8).

## 3. Discussion 

Water deficit not only decreases seeds germination, but also limits seedling growth and development after germination [20]. Previous studies have proved that the seed soaking with chemicals is a cheap and effective technique for the improvement of seed germination and growth under stressful conditions such as drought [21,22]. In this study, seeds soaking with lower concentrations of NaCl (0.5, 1, and 2.5 mM) could significantly increase seed germination and shorten MGS. It was also worthwhile to note that 1 mM of NaCl exhibited pronounced effects on alleviating water stress-induced decreases in GP, GV, VI, and the radicle length of white clover seedlings. The results suggest that seed priming with 1 mM of NaCl could be a simple approach for enhancing tolerance to water stress in white clover. During seeds germination, it is well known that phytohormones, including ABA, GA, IAA, and CTK play critical roles [23]. Gibberellin (GA) and auxin indole-3-acetic acid (IAA) are previously in relation to plants growth. GA promotes plant elongation by increasing the ductility of cell walls, and it also could stimulate amylase activities to induce amylolysis during seed germination. IAA is mainly related to the root growth of seedlings [23,24]. ABA acts as a negative regulator of seed germination, and its accumulation promotes seed dormancy [25]. In this study, NaCl pretreatment significantly increased the content of GA and IAA during seed germination under water stress. The result implied that NaCl-induced seed germination and growth could be associated with the increases in endogenous GA and IAA in white clover under water stress. In addition, the stress-induced ABA accumulation in both of NaCl-treated and untreated seeds could be one of main factors inhibiting the seed germination of white clover. During seed germination, increases in endogenous GA and IAA content due to NaCl pretreatment might be in association with positive starch and antioxidant metabolism contributing to better growth and stress tolerance in white clover, which will be discussed in detail below.

In response to drought stress, plants accumulate organic metabolites such as soluble carbohydrates, proline, etc. and inorganic ions, including Na^+^, K^+^, and Ca^2+^, in order to increase the concentration of cell fluids for the maintenance of osmotic balance. The accumulation of organic osmoregulants also has the function of osmoprection when plants suffer from abiotic stress [14,26]. Amylases are key enzymes that catalyze the hydrolysis of starch through the breakdown of starch into small carbohydrates for seedling growth and OA during seed germination [27]. Our results showed that water stress significantly inhibited amylolysis due to a decrease in β-amylase activity. However, white clover seeds soaking with NaCl improved amylolysis and accumulated more soluble sugars through the maintenance of higher β-amylase activity than untreated seeds during germination under water stress. These obtained results indicate that soluble sugars from NaCl-regulated amylolysis provided sufficient energy for seed germination, and they also could act as osmotic regulators to reduce the osmotic potential contributing to enhanced stress tolerance during the seed germination of white clover. In addition, the greater accumulation of free proline was also observed in NaCl-pretreated seedlings in response to water stress. Proline is one of the most abundant organic osmolytes involved in the maintenance of cell membrane stability and scavenging of superoxide anion and other free radicals in plants under drought stress [28,29,30]. In this study, pretreatment with NaCl significantly increased the accumulation of free proline during seed germination, contributing to better stress tolerance in white clover.

The accumulation of reactive oxygen species (ROS) is one of the most obvious toxic effects when plants respond to drought stress. ROS can oxidize cell membranes and attack organelles, leading to lipid peroxidation, protein degradation, and damage to cell function in plants under abiotic stress [31]. The scavenging of ROS in plants depends on antioxidant systems, including nonenzymatic and enzymatic constituents. SOD, CAT, POD, and key enzymes (APX, DHAR, GR, and MDHR) involved in the ascorbate–glutathione cycle (ASA–GSH cycle) are important antioxidant enzymes for the maintenance of ROS balance. Nonenzymatic antioxidants mainly include intermediate metabolites of ASA–GSH cycle, such as ASA and GSH in plants [32]. SOD can specifically catalyze the disproportionation of O_2_^.−^ into H_2_O_2_ and O_2_. CAT, POD, and the metabolism of the ASA–GSH cycle act as the scavengers of H_2_O_2_ in plant cells [33,34]. In this study, seeds soaking with NaCl significantly enhanced antioxidant defense through the maintenance of higher antioxidant enzymes (SOD, POD, APX, and GR) activities, transcript levels of genes encoding antioxidant enzymes (*FeSOD*, *APX*, and *DHAR*), and the accumulation of antioxidant metabolites (ASA, GSH, and GSSG) during germination in response to water stress. It has been found that exogenous applications of IAA induced significant improvements of endogenous IAA levels, leading to enhanced antioxidant defense systems in white clover under drought stress [35]. IAA-regulated enhancement of antioxidant defense were also observed in other plant species in response to drought stress [36,37]. Our current findings suggest that NaCl-regulated antioxidant defense may be associated with an increase in IAA levels, which is beneficial to better cope with stress-caused oxidative damage during the seed germination of white clover.

Dehydrins, also known as late embryogenesis abundant (LEA) proteins, are hydrophilic proteins with high thermal stabilities in plants. Dehydrins plays an critical role in protecting other cellular proteins and membrane structures from dehydrant damage in germinating seeds and plants under various abiotic stresses [38,39]. Previous study has found that spermine induced dehydrin synthesis in the leaves of white clover in association with better drought tolerance [40]. The inhibition of genes encoding seed-specific dehydrins caused significant decreases in seed germination and the longevity of *Arabidopsis* during storage at low moisture and salt stress [41]. During seed germination, exogenous γ-aminobutyric acid effectively alleviated salt damage and improved growth related to dehydrin accumulation and dehydrin-encoded gene expression in white clover [42]. Our current results showed that transcript levels of *Y2K* and *Y2SK* were significantly up-regulated by water stress, while *dehydrin b* expression was significantly down-regulated under drought stress, implying that different types of dehydrins might have different responses to water stress during seed germination. Interestingly, water stress did not alter the expression level of *SK2* in seedlings without NaCl pretreatment, but seeds soaking with NaCl significantly up-regulated *SK2* expression during germination under water stress. The finding indicates that NaCl-priming-induced stress tolerance could be associated with the upregulation of *SK2* expression during seed germination.

## 4. Materials and Methods

### 4.1. Plant Materials and Treatments

Seeds of white clover (cv. ‘Ladino’) were used as experimental materials. For the priming treatment, seeds were immerged into distilled water for 1 h and then soaked in different concentrations of NaCl solution (0 (control), 0.25, 0.5, 1, 2.5, 5, and 10 mM) for 2 h. After rinsing three time with distilled water, the pretreated seeds were germinated in petri dishes with four sheets of filter paper moistened with distilled water or 19% (*w*/*v*) polyethylene glycol 6000 (PEG 6000) for water stress. The germination rate of seeds is obviously different and easily compared under the condition of 19% PEG. Each petri dish contained 50 seeds, and six independent replicates were set for each treatment. The petri dishes were randomly placed at in growth chamber with a 12 h photoperiod, 700 μmol m^−2^ s^−1^ photosynthetic photon flux density, and 23/19 °C (day/night) for seven days. After seven days of germination, seedlings were sampled for physiological and molecular analyses.

### 4.2. Determination of Seed Germination Characteristics and Starch Metabolism

The GV or GP was evaluated on the third or the seventh day of germination, respectively. GI and MGT were calculated according to the methods of Zhang et al. (2007) [43]. Seedling FW, DW, RL, and VI were measured after seven days of germination. Seedling dry weight was measured after drying at 105 °C for 2 h, and then maintaining at 80 °C for three days. The formula (VI = FW × GI) was used for calculating VI [20]. For starch content analysis, seedlings (0.5 g) were collected and dried in an oven. Dry tissue (0.05 g) with 6 mL ethanol (80%) were placed in 10 mL centrifuge tubes and then extracted in a water bath at 80 °C for 30 min. The mixture was centrifuged at 12,000× *g* for 10 min. The residue was obtained for starch content analysis [44]. The activities of amylase enzymes were measured using the method of Tarrago and Nicolas [45] and Kishorekumar et al. [46]. 

### 4.3. Determination of Endogenous Phytohormones

For extracting ABA, GA, and IAA, 0.4 g of fresh seedlings were ground with 3 mL methanol: isopropanol (1:4, *v*/*v*) with 1% of glacial acetic acid. The mixture was incubated for 1 h at 4 °C in the dark and then centrifuged for 15 min (4 °C and 8000× *g*). The supernatant (2 mL) was dried and then redissolved in 300 μL of methanol. After that, the mixture was filtered through a 0.22 μm poly tetra fluoroethylene (PTFE) filter [47]. Endogenous ABA, GA, and IAA concentration were detected by using Waters Acquity UPLCSCIEX Se-lex ION Triple Quad 5500 System mass spectrometer (Waters, MilfordMA, USA). A total of 5 μL of samples were injected into a loop and loaded onto an Acquity UPLC BEH C18 column (1.7 μm, 50 × 2.1 mm; Waters, Wexford, Ireland) at 40 °C. The CTK content was quantified by the method of the enzyme-linked immunosorbent assay (ELISA), and the assay kit was purchased from Beijing Fang Cheng Biological Technology Co. Ltd., Beijing, China. 

### 4.4. Determination of Soluble Sugars, Amino Acids, Proline, and Osmotic Potential

OP was measured according to the method of Blum [48]. Collected seedlings were immediately soaked in distilled water for 8 h at 4 °C. Seedlings were then took out and blotted dry. Leaf cells sap was used for detecting the osmolarity (c) using an osmometer (Wescor, Logan, UT, USA). The OP was converted according to the formula: MPa = −c × 2.58 × 10^−3^. Free amino acid content was determined spectrophotometrically by using an assay kit (Suzhou Comin Biotechnology Co., Ltd., Suzhou, China). Free proline or soluble sugar content in seedlings was measured according to the method of Bates et al. [49] or Li et al. [40], respectively. 

### 4.5. Determination of Antioxidant Metabolism and Electrolyte Leakage

A 0.2 g weight of fresh seedlings were extracted with 2 mL of extraction solution (50 mM cold phosphate buffer (4 mL, pH 7.8) containing 1% (*w*/*v*) polyvinylpyrrolidone), and then centrifuged for 30 min (12,000× *g* and 4 °C). The supernatant was collected for assays of MDA content and antioxidant enzyme activities. The SOD activity was measured according to the method of Ries [50]. The activity of CAT, POD, APX, MDHR, DHAR, and GR was detected by following the changes in absorbance at 240, 470, 290, 340, 265, and 340 nm, respectively [51]. The method of Bradford [52] or Dhindsa et al. [53] was used for the determination of total protein content and MDA content, respectively. The formation rate of O_2_^−^ was measured at 530 nm by using the sulfanilamide method [54]. H_2_O_2_ was assayed based on the method of potassium iodide at 390 nm [55]. The EL was determined by using a conductivity meter (DDS-307A, Shanghai Precision and Scientific Instrument Co., Ltd., Shanghai, China) [56]. The contents of ASA, DHA, GSH, and GSSG were determined by using assay kits (Suzhou Comin Biotechnology Co., Ltd., China).

### 4.6. Genes Expression Analyses

Real-time quantitative polymerase chain reaction (qRT-PCR) was used for detecting transcript levels of genes. For total RNA, 0.1 g of fresh seedlings were extracted by using an RNeasy Mini Kit (Qiagen). The RNA was reverse-transcribed to cDNA using a revert Aid First Stand cDNA Synthesis Kit (Fermentas). A total of 500 ng RNA was used for each cDNA synthesis. The cDNA was subjected to qPCR using primers of antioxidant enzyme genes (*Cu/ZnSOD*, *FeSOD*, *MnSOD*, *POD*, *CAT*, *APX*, *DHAR*, *CytGR*, and *MDHR*) [57] and dehydrin-encoded genes (*Y2SK*, *Y2K*, and *SK2*) [58] (Table 1). For all genes, PCR conditions were as follows: 5 min at 94 °C, denaturation at 95 °C for 30 s (40 repeats), annealing at 58–64 °C for 30 s, and extension at 72 °C for 30 s (Table 2). The formula 2^−∆∆*C*t^ described by Xia et al. [31] was used for calculating gene transcript levels.

### 4.7. Statistical Analysis

SPSS 20 (IBM, Armonk, NY, USA) was used for analyzing all data. The significant differences among C, C + NaCl, PEG, and PEG + NaCl treatments were tested at *p* ≤ 0.05.

## 5. Conclusions

In summary, seed soaking with NaCl (1 mM) is a cheap and effective approach for improving the germination of white clover seeds under water stress. Exogenous NaCl induced significant increases in endogenous GA and IAA contents, and also improved amylase activities and amylolysis, thereby enhancing the osmotic adjustment, and maintaining growth during seeds germination in response to water stress. NaCl-enhanced antioxidant defense may be associated with an increase in IAA levels, which effectively alleviated the oxidative damage caused by water stress, and stabilized the cell membrane. In addition, NaCl-upregulated *SK2* expression could be another important mechanism of drought tolerance during seeds germination under water stress.

## Figures and Tables

**Table 1:** Effects of seeds soaking with water or NaCl on the germination percentage (GP), germination vigor (GV), germination index (GI), and mean germination speed (MGS) during seven days of germination under normal conditions and water stress induced by 19% polyethylene glycol (PEG) 6000. Different letters in a vertical column indicate significant differences among different NaCl treatments under normal condition or water stress (*p* ≤ 0.05). ±SE of mean (*n* = 6). NC, normal condition; WS water stress.

NaCl (mM)	Germination Percentage		Germination Vigor		Germination Index		Mean Germination Speed	
	NC	WS	NC	WS	NC	WS	NC	WS
0.00	98.0 ± 2.0 a	36.7 ± 3.1 c	92.7 ± 3.1 a	18.0 ± 3.5 d	43.90 ± 1.91 a	6.96 ± 1.35 c	1.31 ± 0.14 a	3.75 ± 0.53 a
0.25	96.7 ± 3.1 a	42.7 ± 5.0 bc	93.0 ± 5.3 a	32.7 ± 2.3 c	44.11 ± 2.56 a	11.44 ± 1.43 bc	1.26 ± 0.11 a	2.46 ± 0.32 b
0.50	97.3 ± 2.3 a	46.0 ± 6.0 b	92.7 ± 5.0 a	40.7 ± 3.1 b	44.81 ± 3.82 a	11.88 ± 1.68 b	1.28 ± 0.19 a	2.26 ± 0.26 b
1.00	98.0 ± 2.0 a	58.7 ± 2.3 a	92.7 ± 1.2 a	47.3 ± 4.6 a	45.43 ± 1.00 a	17.95 ± 2.16 a	1.22 ± 0.02 a	2.09 ± 0.49 b
2.50	97.3 ± 1.2 a	50.0 ± 5.3 b	93.0 ± 2.0 a	40.0 ± 2.0 b	46.31 ± 0.50 a	13.83 ± 2.33 b	1.16 ± 0.08 a	2.44 ± 0.27 b
5.00	96.7 ± 3.1 a	40.7 ± 4.6 bc	93.0 ± 5.3 a	29.3 ± 3.1 c	45.40 ± 2.15 a	10.68 ± 4.97 c	1.15 ± 0.08 a	2.71 ± 0.46 b
10.00	94.0 ± 2.0 a	35.3 ± 2.3 c	91.3 ± 2.3 a	20.7 ± 4.2 d	43.85 ± 0.46 a	6.34 ± 0.86 c	1.22 ± 0.06 a	3.42 ± 0.30 a

**Table 2:** Primer sequences and their corresponding GenBank accession numbers of the analyzed genes.

Target Gene	Accession No.	Forward Primer (5′–3′)	Reverse Primer (5′–3′)	Tm (°C)
*Cu/Zn* *SOD*	JQ321597.1	AACTGTGTACCACGAGGACTTC	AGACTAACAGGTGCTAACAACG	58
*Fe* *SOD*	KP202173	ACACGATTTCTCAGGGTTACGAC	GCGGCCAAGACTATCAGTTCCAT	58
*Mn* *SOD*	JQ321598.1	TAAGGGAACCTACCCGATAACT	CCAGGACCAAACGTCACCAAAG	66
*CAT*	JQ321596.1	AACAGGACGGGAATAGCACG	ACCAGGTTCAGACACGGAGACA	58
*POD*	JQ321606.1	CACTTGGTTTAGTTTTGTCGCC	AACACGGTCTTGTCTGCTACG	64
*APX*	JQ321599.1	TAAAGATAGTCAACCCACCTCAACA	ACCAGTCTTGGGAAACAACGTA	58
*MDHR*	KP202172	CCAACTGCCTAAAGCCACATCT	GAAGAAAGGAAACTAACGGAGCAT	64
*DHAR*	KP202171	TGGTTACCTCCCGACCCTAT	TCTTACCAAGGAACTTTAGTCAGG	58
*CytGR*	JQ321602.1	TAAACTTCCACTCCCTTTCTATCG	CTACAATATGGGTTGAGGACAGGT	58
*Dehydrin b*	GU443960.1	TCCAGTCATCCAGCCTGTTG	CCAGCCACAACACTTGTCA	60
*SK2*	GU443960.1	TGGAACAGGAGTAACAACAGGTGGA	TGCCAGTTGAGAAAGTTGAGGTTGT	58
*Y2K*	JF748410.1	AGCCACGCAACAAGGTTCTAA	TTGAGGATACGGGATGGGTG	60
*Y2SK*	GU443965.1	GTGCGATGGAGATGCTGTTTG	CCTAATCCAACTTCAGGTTCAGC	60
*β* *-Actin*	JF968419	TTACAATGAATTGCGTGTTG	AGAGGACAGCCTGAATGG	58
